# Fungal L-Methionine Biosynthesis Pathway Enzymes and Their Applications in Various Scientific and Commercial Fields

**DOI:** 10.3390/biom14101315

**Published:** 2024-10-17

**Authors:** Kamila Rząd, Aleksandra Kuplińska, Iwona Gabriel

**Affiliations:** Department of Pharmaceutical Technology and Biochemistry, Gdansk University of Technology, 80-233 Gdansk, Poland; kamila.rzad@pg.edu.pl (K.R.); aleksandra.kuplinska@pg.edu.pl (A.K.)

**Keywords:** wine industry, antifungal, L-methionine production, molecular markers, L-methionine biosynthesis

## Abstract

L-methionine (L-Met) is one of the nine proteinogenic amino acids essential for humans since, in human cells, there are no complete pathways for its biosynthesis from simple precursors. L-Met plays a crucial role in cellular function as it is required for proper protein synthesis, acting as an initiator. Additionally, this amino acid participates in various metabolic processes and serves as a precursor for the synthesis of S-adenosylmethionine (AdoMet), which is involved in the methylation of DNA molecules and phospholipids, as well as in maintaining genome stability. Due to its importance, fungal L-methionine biosynthesis pathway enzymes are being intensively studied. This review presents the current state of the art in terms of their cellular function, usefulness as molecular markers, antifungal targets, or industrial approaches.

## 1. Introduction

L-methionine (L-Met) belongs to the aspartate family of amino acids, which are not synthesized by mammalian cells. It is a unique in structure amino acid, which is composed of a thioether hydrophobic side chain. L-Met is crucial for cell functioning as it plays an essential role in the initiation of translation of protein biosynthesis and takes part in various metabolic processes inside the cell [1]. This amino acid is biosynthesized by fungal, bacterial, and plant cells from L-homoserine (L-Hom), but the respective steps of the pathway leading to L-Met production differ across organisms. Due to the extensive biological interdependencies of the L-Met biosynthetic pathway, studying the mechanisms of the enzymes involved in this pathway can provide valuable insights into cellular functions and have applications in various scientific and commercial fields.

Because of the important role that L-Met plays in various organisms and the absence of an L-Met biosynthesis pathway in humans, enzymes involved in L-Met biosynthesis in fungi are considered by researchers as new possible targets for antimicrobial drugs [2]. Another positive aspect is that this amino acid level in human serum stands at only 20 μM [3], which is probably too low to rescue auxotrophic fungal strains incapable of methionine biosynthesis (as has been outlined in subsequent subsections). All of this led to deeper studies about the enzymes involved in the biosynthesis pathway of L-Met, their impact on the virulence of microorganisms, their phenotype, and their ability to grow.

Selected methionine biosynthesis enzymes are also considered as molecular markers. For years, strains of *Saccharomyces cerevisiae* with the deleted gene encoding the bi-functional *O*-acetyl-L-homoserine/*O*-acetyl-L-serine sulfhydrylase enzyme (Met17p), such as BY4741, have been commonly used in laboratories. The deletion of *MET17* has been employed as an auxotrophic marker based on the assumption that this gene is essential for growth in media lacking organosulfur compounds [4]. Using an enzyme from the L-Met biosynthesis pathway in fungi is also an increasingly attractive approach for the commercial production of L-Met, an amino acid of significant industrial importance. Efforts are being made to develop a cost-effective method for producing L-Met from natural sources, without relying on genetically modified organisms [5]. Researchers also indicated that L-Met biosynthesis is connected with the production of intercellular H_2_S, an internal, physiological regulator in various cells, which exerts its effects on various targets and pathways by interacting with proteins, and DNA. Hydrogen sulfide, a volatile sulfur compound produced during yeast fermentation, plays an important role in the wine industry. The formation of H_2_S is correlated with an unpleasant odor in wine, similar to rotten eggs or sewage, and negatively impacts wine quality. The formation of H_2_S is closely related to the L-Met biosynthesis pathway, a route for the metabolism of organic sulfur compounds. The level of hydrogen sulfide in wine should be properly managed during the fermentation, as excess amounts can greatly overwhelm the aroma profile of the wine and reduce its quality and appeal [6].

## 2. L-Methionine Biosynthesis Pathway in Fungal Cells and Its Connections

**Figure 1:**
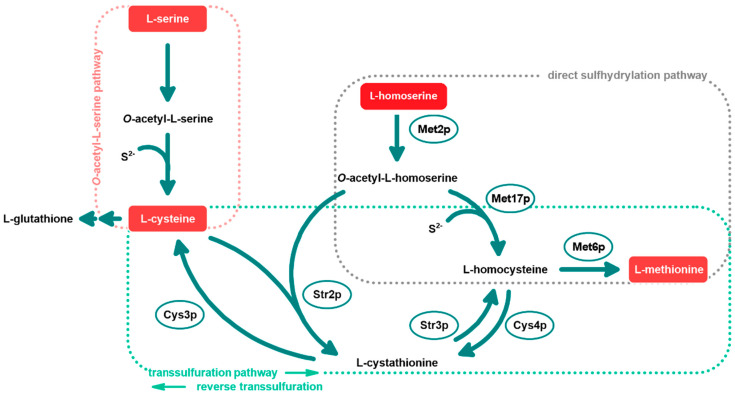
The biosynthesis pathway of L-Met in *S. cerevisiae* cells. Met2p *O*-acetyl-L-homoserine *O*-acetyltransferase (EC 2.3.1.31); Met17p bifunctional *O*-acetyl-L-homoserine/*O*-acetyl-L-serine sulfhydrylase (EC 2.5.1.49, EC 2.5.1.47); Str2p cystathionine γ-synthase (EC 2.5.1.48); Str3p cystathionine β-lyase (EC 4.4.1.8); Cys4p cystathionine β-synthase (EC 4.2.1.22); Cys3p cystathionine γ-lyase (EC 4.4.1.1); Met6p methionine synthase (EC 2.1.1.13).

L-Met is one of the five proteinogenic amino acids constituting the so-called aspartate family (includes L-Asp, L-Asn, L-Met, L-Thr and L-Ile). Biosynthesis of these amino acids starts from the common precursor, i.e., oxaloacetate, which is in the five initial steps converted into L-homoserine. Biosynthesis of methionine from the homoserine occurs in bacteria, fungi, and plants, but its course is not exactly the same in all these organisms [1]. In the main fungal version of methionine biosynthesis, as shown in Figure 1, starting from the branch at the L-Hom intermediate, it is first *O*-acetylated in the reaction catalyzed by L-homoserine transacetylase (Met2p, EC 2.3.1.31).

A sulfur atom is incorporated into *O*-acetyl-L-homoserine in two different ways, either by the direct sulfhydrylation pathway or the transsulfuration pathway. The direct sulfhydrylation pathway utilizes inorganic sulfide for the L-homocysteine production by a bifunctional *O*-acetyl-L-homoserine/*O*-acetyl-L-serine sulfhydrylase enzyme (Met17p, EC 2.5.1.49, EC 2.5.1.47). For *S. cerevisiae* this enzyme is also called Met15p [7]. L-homocysteine corresponds to a crossroads between direct sulfhydrylation and the transsulfuration pathway. The final step of the L-Met biosynthesis involves the methionine synthase (Met6p) enzyme, which, in contrast to the human equivalent, is cobalamin-independent, and utilizes L-homocysteine and 5-methyl-tetrahydrofolate [8]. The synthesis of L-homocysteine via the transsulfuration pathway involves the action of two different enzymes: cystathionine-γ-synthase (Str2p, EC 2.5.1.48) and cystathionine-β-lyase (Str3p, EC 4.4.1.8) which transfer sulfur atom between L-cysteine and L-homocysteine. The Str2p enzyme catalyzes the reaction of the formation of L-cystathionine from *O*-acetyl-L-homoserine and L-cysteine, which is then used by Str3p to yield L-homocysteine. In most fungal species, there also exists a reverse transsulfuration pathway that transforms L-homocysteine to L-cystathionine by cystathionine-β-synthase (Cys4p) followed by the formation of L-cysteine by cystathionine-γ-lyase (Cys3p) (Hébert et al. 2011). The reverse transsulfuration pathway is absent in *Schizosaccharomyces pombe* cells [9]. The L-Met biosynthesis pathway is linked to the pathway of L-cysteine biosynthesis. In contrast to bacterial cells, some fungal organisms, like *Aspergillus nidulans* and *Neurospora crassa*, synthesize L-cysteine in two distinct ways [10]: through the mentioned reverse transsulfuration pathway or the *O*-acetyl-L-serine (OAS) pathway, which begins with L-serine, followed by the reaction between sulfide and *O*-acetyl-L-serine. (Toh-e et al. 2018; de Melo et al. 2019). The OAS pathway is poorly characterized among fungal species. It is known that the OAS pathway is absent in *Candida glabrata* and *S. cerevisiae* species, which means that in these organisms the only functional route for L-Cys biosynthesis is the reverse transsulfuration pathway [11]. Interestingly, *Schizosaccharomyces pombe,* which lacks the reverse transsulfuration pathway, appears to possess two *O*-acetyl-L-homoserine-sulfhydrylases, linking features of filamentous fungi and yeasts [12].

## 3. Characterization of Fungal Enzymes Involved in the L-Methionine Biosynthesis Pathway

### 3.1. L-homoserine O-acetyltransferase (EC 2.3.1.31)

**Figure 2:**
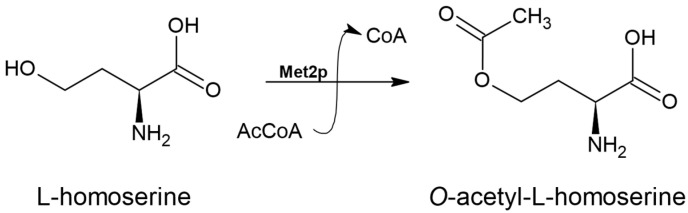
The reaction catalyzed by L-homoserine *O*-acetyltransferase (EC 2.3.1.31).

The first enzyme of the L-Met biosynthesis pathway in fungal cells is L-homoserine *O*-acetyltransferase (Met2p) encoded by the *MET2* gene. Met2p requires L-homoserine (L-Hom) and acetyl coenzyme A (AcCoA) as substrates as it is responsible for the activation of the L-Hom hydroxyl group through its acetylation via a covalent acyl-enzyme intermediate [13] (Figure 2).

Met2p belongs to the α/β hydrolase fold enzyme superfamily, which is characterized by the eight-stranded β-sheet connected by α-helices. The structures of the Met2p enzyme from the Ascomycetes group exhibit a comparable overall folding pattern, characterized by the formation of two distinct domains: a main core domain and a lid domain, which plays a role in the enzyme’s dimerization [14]. The active site of these enzymes is found on a characteristic structural element known as the “nucleophilic elbow” involving a catalytic triad in which a nucleophilic residue may vary between serine, histidine, or aspartic acid [13]. L-homoserine *O*-acetyltransferase possesses a catalytic triad of conserved motif Ser-Asp-X-Leu-Phe, where serine acts as a nucleophilic residue whereas histidine and aspartic acid help to activate serine and take part in enzyme-acyl bond formation [13]. The same catalytic motif was observed for bacterial *Mycobacterium smegmatis* (PDBID: 6IOG), *Haemophilus influenzae* (PDBID: 2B61), and *Leptospira interrogans* (PDBID: 2PL5) as well as L-homoserine *O*-acetyltransferase from *Candida albicans* [15].

A series of studies has shown that homoserine *O*-acetyltransferase activity is essential for the prototrophy of *C. albicans* and *S. cerevisiae* fungi with respect to L-Met [16]. ΔMet2 *S. cerevisiae* mutants additionally exhibited resistance to the presence of methylmercury in the medium. Montoya et al. observed that Δmet2 *Candida guilliermondii* mutants, auxotrophic for L-Met, like the *S. cerevisiae* mutant, form brown colonies on a solid agar medium containing lead, probably due to the presence of a dark lead sulfide coating on the cell surface [17,18]. This team attempted to use this phenomenon as a selective marker in genetic modifications of the *C. guilliermondii* genome. It was demonstrated that the *MET2* knockout strategy is not suitable for effectively disrupting multiple genes. However, the results indicate that the *MET2* knockout cassette works well as a system for disrupting a single gene in *C. guilliermondii* [17].

Studies of Δmet2 *Cryptococcus neoformans* mutants showed avirulence in a mouse infection model via inhalation. As expected, the strain was auxotrophic for L-Met. A screening of a compound library of small molecules for Met2p inhibitors was also conducted, and the compound 6-carbamoyl-3a,4,5,9b-tetrahydro-3H-cyclo-penta[c]quinoline-4-carboxylic acid (CTCQC) was tested. Although CTCQC proved to be an effective in vitro inhibitor of the Met2p enzyme, it did not show antifungal activity against *C. neoformans* in a minimal medium at concentrations up to 128 µg mL^−1^ [19]. Comparable results were observed in studies of the bacterium *Mycobacterium tuberculosis*. The ΔmetA mutant, which has a deleted gene encoding bacterial L-homoserine *O*-acetyltransferase, is auxotrophic for *O*-acetylhomoserine, L-homocysteine, and L-Met. Despite their high levels in serum, these metabolites are not present in sufficient amounts in the host’s preferred niches, or the transport mechanisms for these metabolites in the bacteria may not be active in living organisms. Additionally, the ΔmetA mutant is incapable of proliferating in primary human macrophages and is highly susceptible to elimination in both immunocompetent and immunocompromised mice [20].

Similarly, the rice blast fungus, *Magnaporthe oryzae*, exhibited significantly reduced growth and pathogenicity following *MET2* gene disruption compared to the wild type. Li et al. [21] additionally demonstrated that transgenic rice cultivars modified using the Host-Induced Gene Silencing (HIGS) technique to target the genes *MET2* and *CYS2* (homologs encoding proteins with L-homoserine *O*-acetyltransferase activity) exhibited strong resistance to the pathogen. They prepared a siRNA construct capable of silencing the expression of *MET2* and *CYS4* in *M. oryzae* and introduced it into transgenic plants, demonstrating both that L-homoserine *O*-acetyltransferase could be a potential target for novel fungicides and that HIGS could be an effective strategy for controlling rice blast [21].

**Figure 3:**
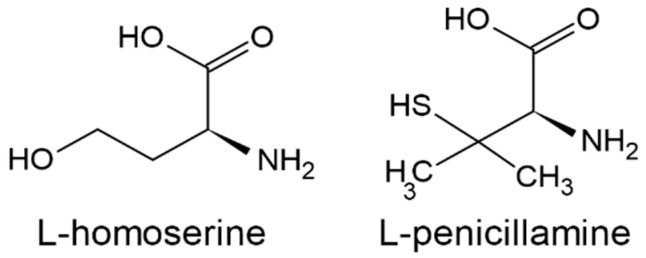
Structures of L-homoserine and its analog L-penicillamine, an inhibitor of *C. albicans* Met2p.

These results indicate that L-homoserine *O*-acetyltransferase might be considered a great potential molecular target for antimicrobial chemotherapy [19]. In this regard, some structural analogs of L-homoserine were tested as Met2p inhibitors. Moderate inhibitory potential against Met2p from *C. albicans* was observed for L- and D-penicillamine [15] (Figure 3) This effect was related to antifungal activity. The minimal inhibitory concentrations (MIC_50_) at which growth is inhibited by 50% ranged from 32 to 1024 µg mL^−1^ for several fungal strains. For *C. glabrata* and *S. cerevisiae*, this value depends on the availability of L-Met in the medium, confirming that the molecular target is connected with the L-Met biosynthesis pathway.

### 3.2. O-acetyl-L-homoserine Sulfhydrylase Enzyme (EC 2.5.1.49)

**Figure 4:**
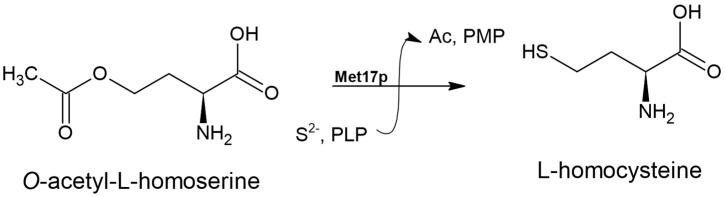
The reaction catalyzed by the bi-functional *O*-acetyl-L-homoserine/*O*-acetyl-L-serine sulfhydrylase enzyme (EC 2.5.1.49, EC 2.5.1.47) from *S. cerevisiae*. ‘Ac’ denotes acetate.

The *O*-acetyl-L-homoserine sulfhydrylase EC 2.5.1.49 is a PLP-dependent enzyme that uses *O*-acetyl-L-homoserine and a sulfide ion to catalyze the synthesis of L-homocysteine (L-HCT) (Figure 4), a reaction that plays a role in the direct sulfhydrylation pathway of the L-Met biosynthesis pathway [22,23].

In some fungi, an enzyme exists as a bi-functional *O*-acetyl-L-homoserine/*O*-acetyl-L-serine sulfhydrylase (EC 2.5.1.49, EC 2.5.1.47). One of the roles of this enzyme is to produce L-Cys from *O*-acetyl-L-serine (L-OAS) as a part of the transsulfuration pathway [22,24]. The bi-functional enzyme can be found in fungal cells such as *S. cerevisiae*, *Kluyveromyces lactis*, *Yarrowia lipolytica*, *S. pombe, Trichosporon cutaneum* and *A. nidulans*.

The bi-functional *O*-acetyl-L-homoserine/*O*-acetyl-L-serine sulfhydrylase enzyme from *S. cerevisiae* (Met17p) and bacterial *O*-acetyl-L-homoserine sulfhydrylase (Met15p) enzyme from *Wolinella succinogenes* have been analyzed in their crystal structures, showing that they are part of the PLP-dependent Cys/Met enzyme family [25,26]. The activity of this bi-functional enzyme (called Met17p in yeast) is essential for the proper functioning of *S. cerevisiae* cells. ΔMet17 mutants are auxotrophic for L-Met and sulfur, which can be mainly attributed to the toxic accumulation of hydrogen sulfide gas. In contrast, *K. lactis* yeast mutants lacking the gene encoding this enzyme grow more slowly compared to the wild-type strain and exhibit lower levels of sulfur-containing amino acids [24]. The content of sulfur-containing amino acids is changing also in *T. cutaneum* mutants but does not influence its growth rate. The enzyme is physiologically important, though not indispensable. The lack of activity of this enzyme in *Y. lipolytica* and *S. pombe* does not have a physiological impact, in contrast to the bacterial *O*-acetyl-L-homoserine sulfhydrylase enzyme, which is crucial for the virulence rate and survival of *Streptococcus suis* in mouse infection model [27]. The bi-functional *O*-acetyl-L-homoserine/*O*-acetyl-L-serine sulfhydrylase enzymes from *Y. lipolytica*, *S. pombe*, and *T. cutaneum* are probably mainly responsible for recycling the methylthio group from 5′-methylthioadenosine, a byproduct of polyamine synthesis from AdoMet [28]. This role was proved for enzymes from *A. nidulans* [28].

The *S. cerevisiae* Δmet17 mutant has been also used for years as a model organism equipped with an auxotrophic marker for a wide range of studies. However, it has been shown that the Met17p protein is not essential for the survival of *S. cerevisiae* in the absence of exogenous sulfur-containing organic compounds; the mutants are able to utilize an alternative homocysteine biosynthesis pathway involving the enzyme encoded by HSU1 (Yll058W). This enzyme can catalyze the reaction typically carried out by Met17p, albeit with lower efficiency [4,29,30].

Moreover, the bi-functional *O*-acetyl-L-homoserine/*O*-acetyl-L-serine sulfhydrylase enzyme from *S. cerevisiae* (Met17p) has attracted scientific interest due to its commercial potential. A method has been developed for L-Met production from *O*-acetyl-homoserine and 3-methylthiopropionaldehyde using recombinant Met17p purified from *Escherichia coli* BL21-Met17 [5]. L-Met is an amino acid whose commercial use is continually increasing. It is used as a flavoring agent in food additives, in therapies for liver diseases, as a nutritional component in infant formulas, parenteral nutrition, and in sports supplements [31]. The production of L-Met using recombinant Met17p addresses issues such as the formation of racemic D- and L-Met mixtures, chemical contamination of the final product associated with chemical synthesis, and the low yield and difficult purification processes observed in microbial fermentation methods [5]. Production of L-Met in a one-pot process using a combined method of fermentation and biocatalysis was reported by Zhu et al. in 2021. Mutant strains of *E. coli* and homoserine *O*-succinyltransferase enzyme were employed for efficient large-scale production. In contrast to the method described above, Zhu team used homoserine *O*-succinyltransferase which is a bacterial analog of Met2p, an enzyme involved in the L-methionine biosynthesis pathway in *E. coli* [32]. The Met17p enzyme from *S. cerevisiae* may also be used for the production of L-methionine analogs and organic thiols. Most of these compounds are not commercially available but are necessary for synthesizing S-adenosyl-L-methionine analogs [25]. The clinical applications of AdoMet include treatment of depression, osteoarthritis and alcoholic liver disease [33]. Moreover, AdoMet and its analogs are used in research investigating methyltransferases and the regulation of intracellular processes [34,35]. The discovery of novel AdoMet-dependent methyltransferases enables the industrial biosynthesis of various natural products in heterologous hosts [36].

In the wine industry, the aroma and bouquet of wine are of immense importance and directly impact economic gains. Excess H_2_S produced during the yeast fermentation process can lead to off-flavors reminiscent of rotten eggs, which significantly detracts from the quality of the wine. It was found that early-stage H_2_S production positively affects the aroma and bouquet of wine due to subsequent transformations of this compound into more desirable forms [37]. Enzymes Met2p and Met17p from *S. cerevisiae* were evaluated for their impact on the amount of H_2_S produced during fermentation. However, the *S. cerevisiae* Δmet17 and Δmet2 mutants produced higher levels of H_2_S in the later stages of fermentation [6].

### 3.3. Cystathionine-γ-synthase (EC 2.5.1.48)

**Figure 5:**
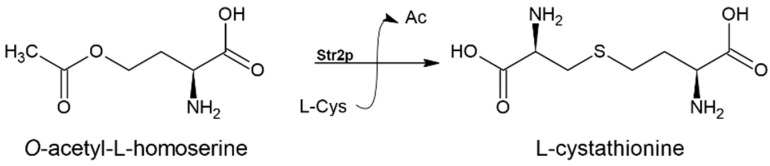
The reaction catalyzed by cystathionine-γ-synthase (EC 2.5.1.48). ‘Ac’ denotes acetate.

Incorporation of a sulfur atom into L-homocysteine can alternatively be carried out via the transsulfuration pathway in the presence of L-cysteine (Figure 1). The first step of the pathway during which L-cystathionine origins from L-cysteine and *O*-acetyl-L-homoserine (an activated form of L-Hom) is catalyzed by the cystathionine-γ-synthase (Figure 5).

Microorganisms activate L-Hom in different ways depending on the organism. Generally, in fungi by creating *O*-acetyl-L-homoserine, in bacteria *O*-succinyl-L-homoserine and in plants *O*-phospho-L-homoserine [38].

Many fungal species like *N. crassa* possess both Str2p and Met17p enzymes; however, Met17p was discovered not to be essential in L-Met biosynthesis in *N. crassa* cells [39]. Deletion of genes encoding Str2p resulted in auxotrophic growth towards L-Met, whereas deletion of Met17p encoding genes did not cause similar results.

Research on *Botrytis cinerea* (grey mold) has highlighted the significant role of the Str2p protein in fungal physiology, including growth, virulence, and response to environmental stresses. The Δstr2 mutant, which is auxotrophic for L-Met, displayed increased sensitivity to the fungicides fluazinam and fludioxonil. Additionally, disruption of the *STR2* gene led to reduced conidia production and defects in sclerotia formation, which may impact its ability to survive. The Δstr2 mutant also showed heightened sensitivity to oxidative stress caused by H_2_O_2_, a reactive oxygen species produced by host plants that can result in substantial cellular damage, as well as to osmotic stress, which affects its growth in plant tissues [40].

### 3.4. Cystathionine-β-lyase (EC 4.4.1.8)

**Figure 6:**
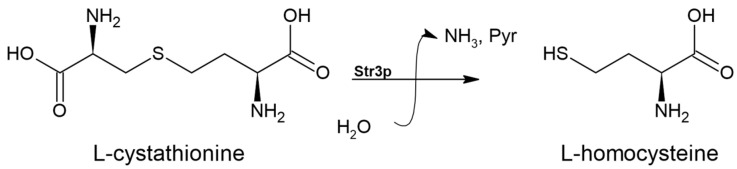
The reaction catalyzed by cystathionine β-lyase (EC 4.4.1.8). ‘Pyr’ denotes pyruvate.

The second step of the transsulfuration pathway, during which L-cystathionine cleaves to L-homocysteine, is catalyzed by cystathionine β-lyase [41]. This reaction is shown in Figure 6.

Both cystathionine γ-synthase and cystathionine β-lyase are members of the PLP-dependent enzyme family and possess a homotetrameric structure. They share similarities in their overall architecture and the arrangement of main active-site residues, including an arginine that recognizes the carboxyl group of the substrate, a tyrosine associated with the PLP cofactor, and a lysine that is likely to collect a proton. Despite these similarities, their catalytic mechanisms differ significantly. Cystathionine β-lyase catalyzes a step in methionine biosynthesis and transfers the proton to L-homocysteine [38].

In *A. nidulans* or *Fusarium graminearum,* deletion of the gene responsible for encoding this enzyme causes an auxotrophy to L-Met [42]. A similar effect was observed for the mutant *Salmonella gallinarum*, a bacterium that is solely responsible for avian typhus [43]. The *METC* gene (encoding cystathionine β-lyase) deletion in *S. gallinarum* resulted in the inability to grow on a minimal medium without L-Met. The ΔmetC mutant was unable to kill chickens after oral inoculation, whereas only 10% of animals inoculated with the wild-type strain survived. The experiment showed that the mutant was not able to grow in the internal organs of the chickens, including the liver or spleen [43]. The virulence of *Salmonella typhimurium* mutant was also impaired in a mouse model of systemic infection in vivo [43].

### 3.5. Cystathionine β-synthase (EC 4.2.1.22)

**Figure 7:**
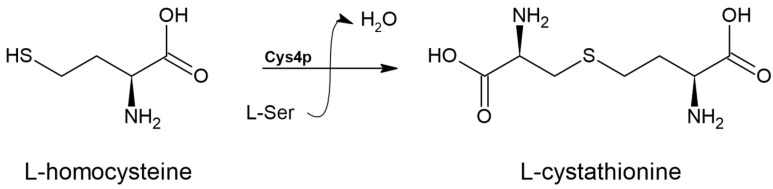
The reaction catalyzed by cystathionine β-synthase (EC 4.2.1.22).

Many organisms can engage in a reverse transsulfuration pathway, where L-homocysteine is first transformed into L-cystathionine and subsequently into L-cysteine [44] (Figure 1). The initial step of this process is mediated by the enzyme cystathionine β-synthase (EC 4.2.1.22) (Figure 7).

**Figure 8:**
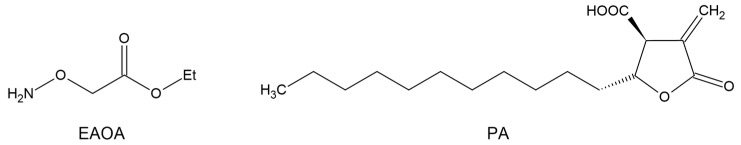
Structures of EAOA and PA, inhibitors of Cy4p from *C. albicans* [45].

Cystathionine β-synthase from *C. albicans* was identified as the key enzyme involved in the intracellular synthesis of H_2_S, an antioxidant molecule that plays a role in various host cell colonization strategies and is secreted to protect against the host’s immune response. Yeast cells with a deletion of the *CYS4* gene exhibited growth defects closely linked to fungal pathogenicity. Additionally, the ability of Δcys4 mutants to form biofilms and hyphae was impaired. This modification also affected protein synthesis, folding, and mannosylation, and caused irregular respiration or mitochondrial dysfunction [45]. Chang et al. elucidated the crystal structure of the catalytic core of Cys4p from *C. albicans* (PDB: 7XRQ) and tested potent inhibitors of Cys4p from *C. albicans*: PA compound (isolated from an edible lichen, along with alternariol and usnic acid) and synthetic ethyl 2-(aminooxy)acetate (EAOA) (Figure 8). Both compounds inhibited fungal growth (*C. albicans*, *C. glabrata*, *Candida parapsilosis*, and *Candida tropicalis*) with MIC values of 32 and 64 µg mL^−1^ for EAOA and PA, respectively. EAOA and PA reduced the H_2_S generation of *C. albicans*, whereas PA more potently inhibited Cys4p than Cys3p and exhibited enzyme selectivity towards fungal Cys4p enzyme [45].

*Aspergillus fumigatus MECA* (gene encoding cystathionine β-synthase) deletion mutant was more sensitive to H_2_O_2_, the cell wall stressor SDS, and the antifungal agent fludioxonil. Furthermore, Sueiro-Olivares et al. demonstrated that the activity of cystathionine β-synthase and cystathionine γ-lyase is essential for survival [46].

It was reported that cystathionine β-synthases from the medicinal mushrooms *Ganoderma lucidum* and *Lignosus rhinocerus* possess angiotensin-converting enzyme (ACE) inhibitory activities. It is worth mentioning that ACE is responsible for blood pressure regulation and is associated with several diseases, such as Alzheimer’s, fibrosis, and lung injury [47]. Moreover, the activity of cystathionine β-synthase from the mushroom *G. lucidum* is linked to its cellulolytic ability and increases the adaptive capacity of this fungus [48]. The ability to break down cellulose is valuable in industries focused on biomass conversion. Additionally, enhancing the growth and adaptive capacity of *G. lucidum* could be beneficial due to its medicinal properties.

### 3.6. Cystathionine γ-lyase (EC 4.4.1.1)

**Figure 9:**
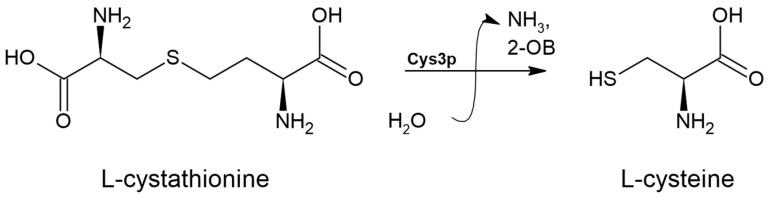
The L-cystathionine conversion reaction catalyzed by cystathionine γ-lyase (EC4.4.1.1). ‘2-OB’ denotes 2-oxybutanoate.

The further conversion of L-cystathionine to L-cysteine through reverse transsulfuration is mediated by the enzyme cystathionine γ-lyase (EC 4.4.1.1). This enzyme is a member of the PLP-dependent enzyme family involved in cysteine and methionine metabolism, which also includes cystathionine γ-synthase, cystathionine β-lyase, and cystathionine β-synthase [49]. The cystathionine γ-lyase enzyme facilitates the transformation of L-cystathionine into L-cysteine, producing α-ketobutyrate and ammonia in the process, as illustrated in Figure 9.

Interestingly, the cystathionine γ-synthase from yeast or human sources has an active site that is nearly identical to that of the enzyme from *E. coli* [50]. The γ-synthase and γ-lyase activities of both enzymes depend on their position within the metabolic pathway and the availability of substrates [50].

It has been shown that *S. cerevisiae* cystathionine γ-lyase is able to convert S-propargyl-cysteine to H_2_S, a relevant gaseous signaling molecule that participated in numerous biological functions. Increasing the intracellular level of H_2_S led to enhanced expression of genes involved in sulfur amino acid biosynthesis and a significant increase in fungal growth [51]. Interestingly, it has been suggested that targeting the endogenous production of this gasotransmitter could be an effective approach to combat these pathogens. The production of H_2_S is also extensively studied in the wine industry to reduce the production of this gas during the fermentation process. The proteins Cys3p and Cys4p have been identified as participating in the H_2_S releasement. However, it has been shown that disruption of the genes encoding *CYS3* and *CYS4* from the *S. cerevisiae* genome did not reduce the levels of H_2_S in vivo [52]. Protein persulfidation (also called sulfhydration) of fungal and host proteins is closely related to the adaptation of *A. fumigatus* to its host and the host’s defense against this pathogen. Deleting the gene encoding cystathionine γ-lyase in fungal cells reduces the rate of persulfidation, a post-translational modification that modulates the activities of peroxiredoxin and alcohol dehydrogenase, both of which are associated with the pathogenic potential of *A. fumigatus*. Additionally, conidia of the mutant strain exhibited significantly higher susceptibility to killing by murine and human macrophage cell lines compared to the wild-type conidia and also displayed reduced virulence in a leukopenic mouse infection model. The *A. fimugatus* mutant lacking the cystathionine γ-lyase gene was also more sensitive to menadione, the thiol-oxidizing drug diamide, and H_2_O_2_. Furthermore, the proper activity of human cystathionine γ-lyase determines the level of persulfidation in host proteins, essential for maximizing the antifungal action of lung-resident alveolar macrophages and epithelial cells [46].

The protein Cys3p was also studied as a potential biological diagnostic target for invasive candidiasis; however, due to its high homology with cystathionine-β-lyase from *Chlamydia*, it cannot be considered a good biomarker [53].

This enzyme may also have commercial potential, as it has been shown that cystathionine γ-lyase from the *Gymnopilus dilepis* mushroom is positively correlated with the biosynthesis of psilocybin, a potential antidepressant alkaloid [54].

### 3.7. Methionine Synthase (EC 2.1.1.13)

**Figure 10:**
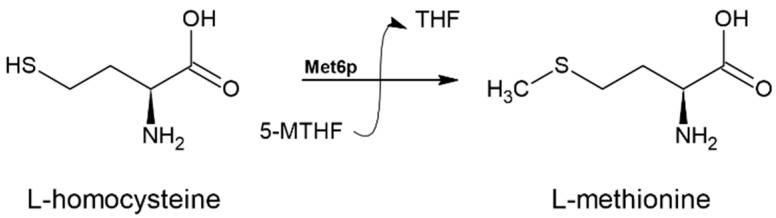
The reaction catalyzed by methionine synthase (EC 2.1.1.13). 5-MTHF and THF denote 5-methyltetrahydrofolate and tetrahydrofolate, respectively.

In fungal microorganisms, the last step of the L-Met biosynthesis pathway is carried out by methionine synthase, which is encoded by the *MET6* gene [44]. This enzyme transfers a methyl group from 5-methyltetrahydrofolate to L-homocysteine, resulting in the production of L-Met and tetrahydrofolate (THF) [55] (Figure 10).

It is important to note that there are two distinct classes of methionine synthases: cobalamin-dependent and cobalamin-independent. Mammalian methionine synthase, which is dependent on cobalamin, functions as an intermediate methyl carrier and plays a crucial role in L-Met biosynthesis [8,56]. In contrast, fungal enzymes, including those from *C. albicans*, do not require cobalamin and instead use 5-methyl-THF as a methyl donor. This difference in cofactor requirement leads to mechanistic variations between the two enzyme types. Cobalamin-independent methionine synthases are also found in some bacteria and higher plants, whereas organisms that cannot produce or obtain cobalamin rely on this type of enzyme [55].

Research revealed that in fungi like *Pichia pastoris* and *C. albicans*, Met6p is situated in the cell nucleus, whereas in *S. cerevisiae*, this enzyme is present in the cytosol [55]. The variations in structure and function between methionine synthases in humans and fungi highlight Met6p as a promising target for antifungal drug development [8]. The gene *MET6* is essential for *C. albicans* viability as colonies of double deletion mutant did not grow even when provided with additional exogenous methionine [8]. Furthermore, evidence suggests that methionine synthase activity may play a crucial role in the virulence of bloodstream-isolated *C. albicans*. The levels of L-homocysteine and 5-methyltetrahydrofolate were elevated in invasive isolates or those that formed a high biofilm [57].

Pascon et al. [58] showed that mutant Δmet6 *C. neoformans* did not exhibit pathogenicity in the inhalation mouse model, displayed increased sensitivity to fluconazole and cyclosporin A and showed slowed growth, auxotrophy for L-Met and a defect in capsule formation (a virulence factor), whereas in *A fumigatus*, the gene responsible for encoding methionine synthase is considered essential for the invasion of host cells [44,59]. The lack of methionine synthase activity in this pathogen causes a metabolic disruption, resulting in decreased intracellular ATP levels, which inhibits fungal growth despite the presence of methionine [60]. Similarly, in the plant pathogen *F. graminearum*, deactivating the MSY1 gene encoding methionine synthase results in aerial hyphal growth defects and reduction in virulence followed by L-Met auxotrophy [42]. Another plant pathogen, *M. oryzae*, becomes avirulent after the deletion of the *MET6* gene. ΔMet6 mutants were auxotrophic for L-Met; even when grown with abundant methionine, these mutants exhibited developmental issues, including decreased pigmentation of the mycelium, impaired formation of aerial hyphae, and reduced sporulation [61].

Studies aimed at improving the sensitivity of diagnostic tests for invasive candidiasis were also conducted using Met6p. Immunoenzymatic tests were performed to detect antibodies against various *C. albicans* proteins [62,63]. In immunocompromised patients, antibodies against Met6p proved to be the most effective biomarker [62].

Methionine synthase is widely present, but the versions found in humans and fungi differ significantly in their structure and function. Additionally, Met6p is exposed on the surface of *C. albicans* cells. These two facts were utilized to develop an effective vaccine protecting against disseminated *C. albicans* by targeting epitopes derived from fructose bisphosphate aldolase and methionine synthase [64]. It has also been shown that survival rates in mice infected with *C. tropicalis*, *C. glabrata*, and *C. albicans* were significantly improved when treated with dendritic cells pulsed with Met6p [65].

The interest in methionine synthase as a potential molecular target for antimicrobial drugs stems from the fact that inhibiting this enzyme causes the accumulation of toxic homocysteine. This accumulation leads to the formation of reactive homocysteine thiolactone, which interferes with the biosynthesis of ergosterol, an important component of fungal cell membranes [3,44]. Despite these promising results, no effective methionine synthase inhibitors with antifungal activity have yet been described [44].

## 4. Conclusions

Methionine and sulfur metabolism play critical roles in the growth and development of fungal cells. Studies concerning the methionine biosynthesis pathway and its connections with cysteine metabolism performed so far mainly focused on usefulness as molecular targets for antifungal agents (Table 1). The investigated pathway differs considerably between pathogens, and even the genes studied within one pathway are often not the same or, in some cases, even not present. The main strategy for using enzymes from this pathway appears to be that of targeting processes that are essential for fungal viability due to antifungal drug development. Although a few sources have reported that some methionine auxotrophs are non-pathogenic, others revealed that disruption of a homologous gene in other pathogenic fungi has no effect on virulence. Despite this, the strong relationship of this pathway with others has allowed the identification of effective enzyme inhibitors with antifungal activity. It is also worth mentioning that selected L-Met biosynthesis enzymes are important for pathogen–host interactions. Another approach related to the methionine biosynthesis pathway is the industrial potential of selected enzymes. Apart from the presence of selected genes’ usefulness as diagnostic markers and auxotrophic mutant strains’ utility as selection markers, a new biotechnological method for L-Met production using recombinant Met17p enzyme from the *S. cerevisiae* has been developed. This enzyme may also be used for the synthesis of commercially important L-methionine analogs and organic thiols. Moreover, the relevance of the yeast methionine biosynthesis pathway enzymes is investigated in relation to the organoleptic properties of food products produced by fermentation, such as wine. Studies performed so far aimed to reduce the production of intercellular H_2_S during the fermentation process.

Overall, the search for antifungal drug candidates among L-Met biosynthetic pathway enzyme inhibitors is undoubtedly worth considering. Moreover, with a better understanding of enzymes’ physiological functions and implications in sulfur metabolism, it is highly probable to discover new potential applications in industry.

## Figures and Tables

**Table 1:** Summary of enzymes application.

Enzyme		Application
**Met2p**	L-homoserine *O* acetyltransferase	The *MET2* knockout cassette is a system for disrupting a single gene in *C. guilliermondii* [17] Δmet2 *C. neoformans* mutants showed avirulence in a mouse infection model via inhalation [19] Δmet2 *M. oryzae* mutants showed reduced growth and pathogenicity [21] *C. albicans* Met2p inhibitor, L-penicillamine, showed antifungal activity [15]
**Met15/Met17p**	bi-functional *O*-acetyl-L-homoserine/*O*-acetyl-L-serine sulfhydrylase	*K. lactis* deletion mutant grows slowly [24] *A.nidulans* enzyme is responsible for recycling the methylthio group from 5′-methylthioadenosine, a byproduct of polyamine synthesis from S-adenosyl-L-methionine [28] Recombinant *S. cerevisiae* Met17p purified from *Escherichia coli* BL21-Met17 has been developed for L-Met commercial production [5] Met17p enzyme from *S. cerevisiae* may also be used for the production of L-methionine analogs and organic thiols [25]
**Str2p**	cystathionine-γ-synthase	Δstr2 mutant of *Botrytis cinerea* displayed increased sensitivity to the fungicides, is less virulence, showed heightened sensitivity to oxidative stress as well as to osmotic stress [40]
**Cys4p**	cystathionine β- synthase	Δcys4 mutant of *C. albicans* exhibited growth defects closely linked to fungal pathogenicity, defect in forming biofilms and hyphae, protein synthesis, folding, mannosylation, and irregular respiration or mitochondrial dysfunction [45] *C. albicans* Cys4p inhibitors, PA and EAOA, showed antifungal activity [45] Enzymes from the medicinal mushrooms *Ganoderma lucidum* and *Lignosus rhinocerus* are potent inhibitors of angiotensin-converting human enzyme [47] Enzyme from *G. lucidum* is linked to its cellulolytic ability and increases the adaptive capacity of this fungus [48]
**Cys3p**	cystathionine γ-lyase	Cys3p from *S. cerevisiae* is capable of converting S-propargyl-cysteine to H_2_S [51] Deleting the gene encoding cystathionine γ-lyase in *A. fumigatus* reduces the fungus’s virulence [46] Enzyme from the *Gymnopilus dilepis* mushroom is positively correlated with the biosynthesis of psilocybin, a potential antidepressant alkaloid [54]
**Met6p**	methionine synthase	Met6p is essential for *C. albicans* viability and may play a crucial role in the virulence of bloodstream-isolated *C. albicans* [8,57] Mutant Δmet6 *C. neoformans* did not exhibit pathogenicity in the inhalation mouse model, displayed increased sensitivity to fluconazole and cyclosporin A, showed slowed growth, and a defect in capsule formation [58] *A fumigatus* methionine synthase is considered essential for the invasion of host cells and growth [44,59] *F. graminearum* with deactivating gene encoding methionine synthase has aerial hyphal growth defects and reduction in virulence [42] Δmet6 mutant of *M. oryzae* is avirulent, has decreased pigmentation of the mycelium, impaired formation of aerial hyphae, and reduced sporulation [61] Antibodies against Met6p proved to be the effective biomarker for *C. albicans* detection Targeting epitopes derived from fructose bisphosphate aldolase and methionine synthase were utilized to develop an effective vaccine protecting against disseminated *C. albicans* [64] Survival rates in mice infected with *C. tropicalis*, *C. glabrata*, and *C. albicans* were significantly improved when treated with dendritic cells pulsed with Met6p [65]
